# Supplementary figures and images for: The budding yeast Fkh1 Forkhead associated (FHA) domain promotes a G1-chromatin state and the activity of chromosomal DNA replication origins
Source: PLoS Genet. 2024 Aug 5;20(8):e1011366. doi: 10.1371/journal.pgen.1011366 (PMC11326605; doi:10.1371/journal.pgen.1011366)

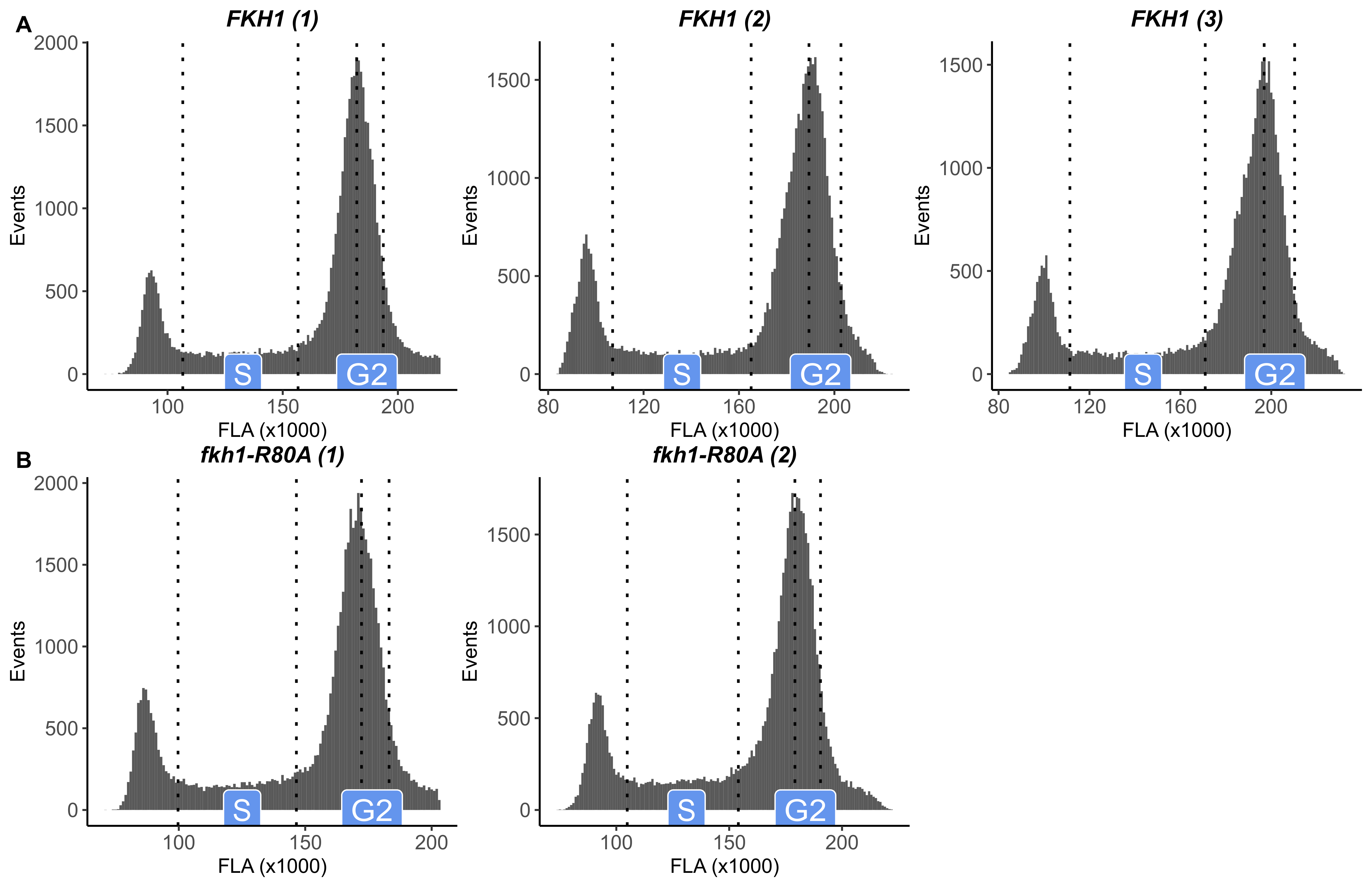

Supplement: S1 Fig — The cell density histograms from the proliferating yeast population as a function of fluorescence are shown for (A) Three independent FKH1 yeast and (B) Two independent fkh1-R80A. Vertical dotted lines indicate the gates used for the collection of the S-phase and G2-phase populations that were processed for sequencing. (TIFF) [file pgen.1011366.s001.tiff]

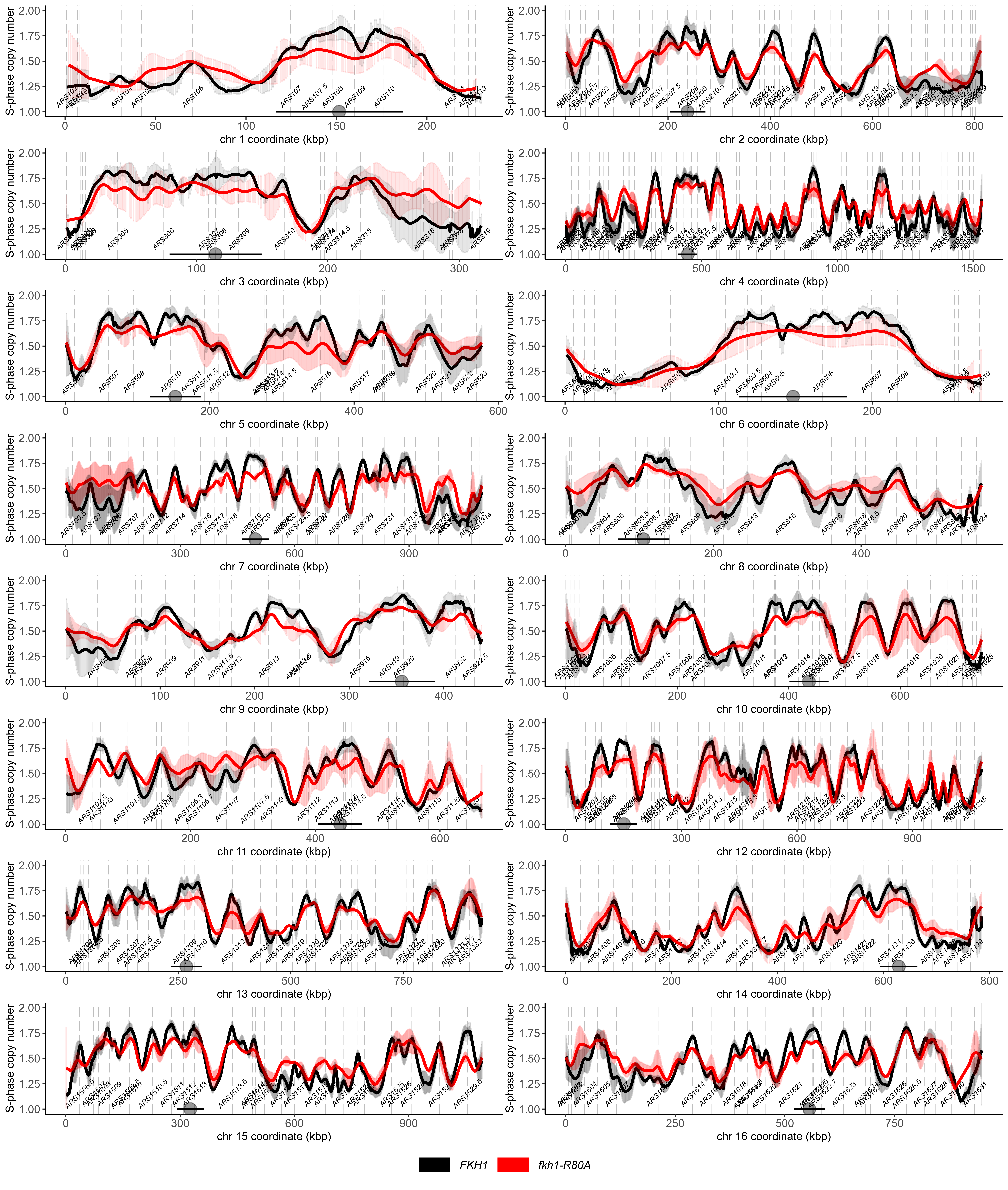

Supplement: S2 Fig — Please see text and Figure legend sections relevant to main text Fig 1A. (TIFF) [file pgen.1011366.s002.tiff]

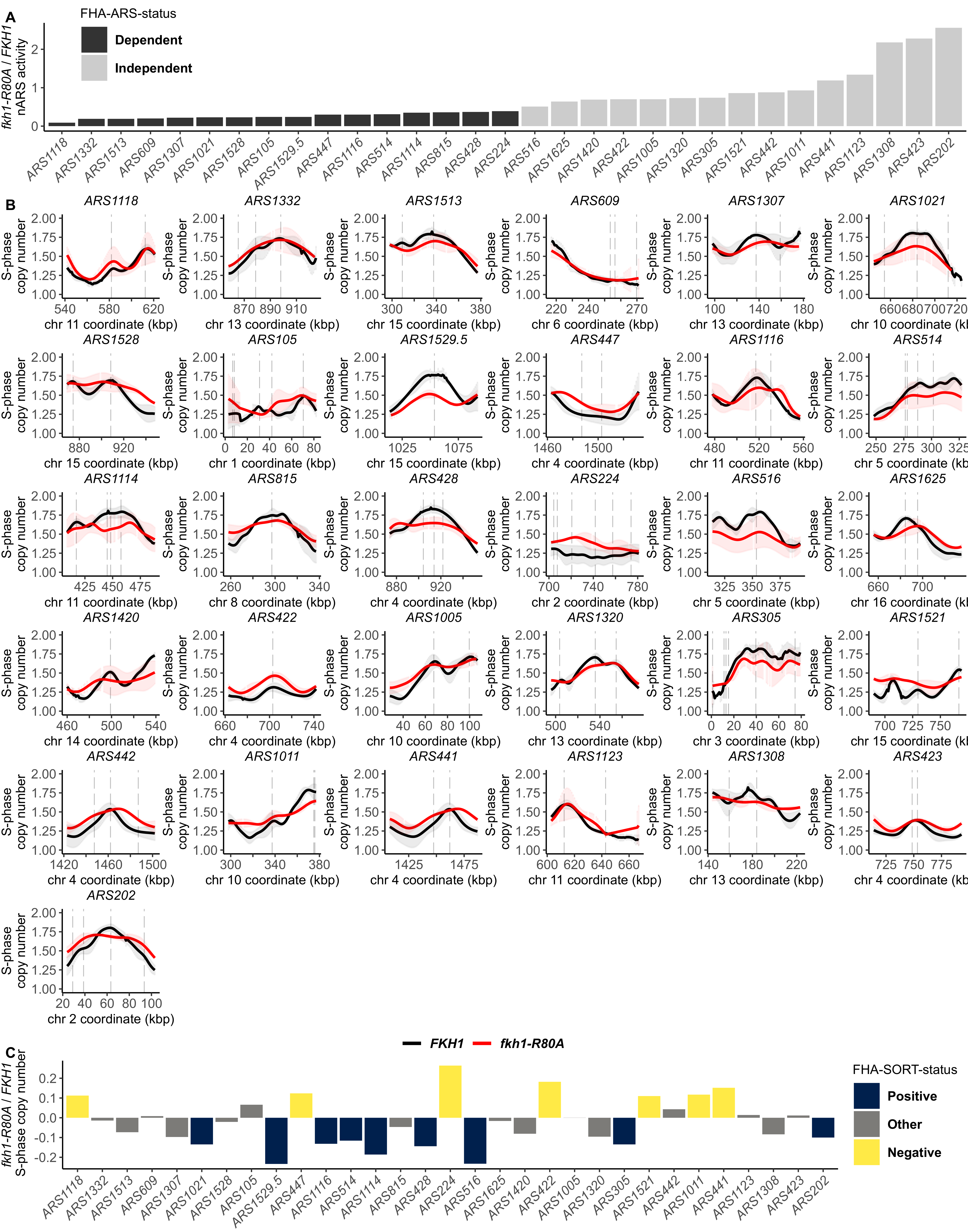

Supplement: S3 Fig — (A) Ratio of ARS activity in fkh1-R80A cells to FKH1 cells (y-axis) for indicated origins (xaxis). FHA-ARS-dependent origins indicated by black bars while FHA-ARS-independent origins indicated by grey bars. (B) Close-up of S-phase copy number scans for FHA-ARS-regulated origins in FKH1 (black) and fkh1-R80A (red) cells. (C) The fkh1-R80A/FKH1 S-phase copy number ratio determined for each FHA-ARS regulated origin color coded based on its FHA-SORT assigned status. FHA-ARS-regulated origins were defined initially based on ORC-origin binding mechanisms and subsequently parsed by ARS activity. Neither ARS609 nor ARS224, each an FHA-ARS-dependent origin, qualified as FHA-SORT-positive. Note that neither origin is active even in FKH1 cells on the chromosome. Thus ORC-origin binding mechanisms might not play a major role in their chromosomal activity, yet might have a large impact on origin activity in a more isolated situation, such as on a plasmid. (TIFF) [file pgen.1011366.s003.tiff]

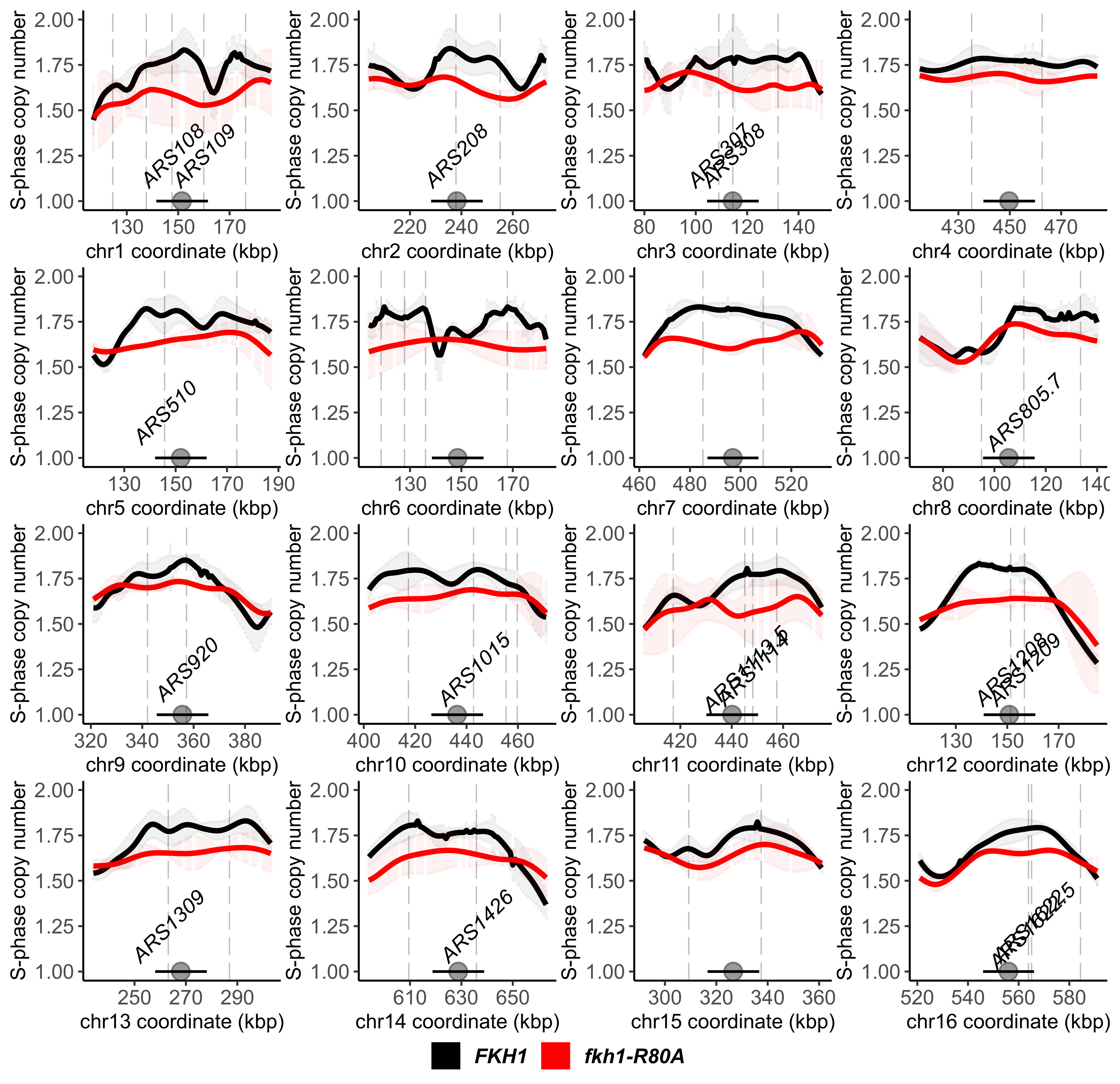

Supplement: S4 Fig — Based on the stringent definition of Cen-associated origins used here, CEN4, CEN6, CEN7, and CEN15 do not contain CEN-associated origins per our definition. Copy numbers are presented as means and 95% confidence intervals from three FKH1 (black) and two fkh1-R80A (red) replicates, as discussed in the main text. (TIFF) [file pgen.1011366.s004.tiff]

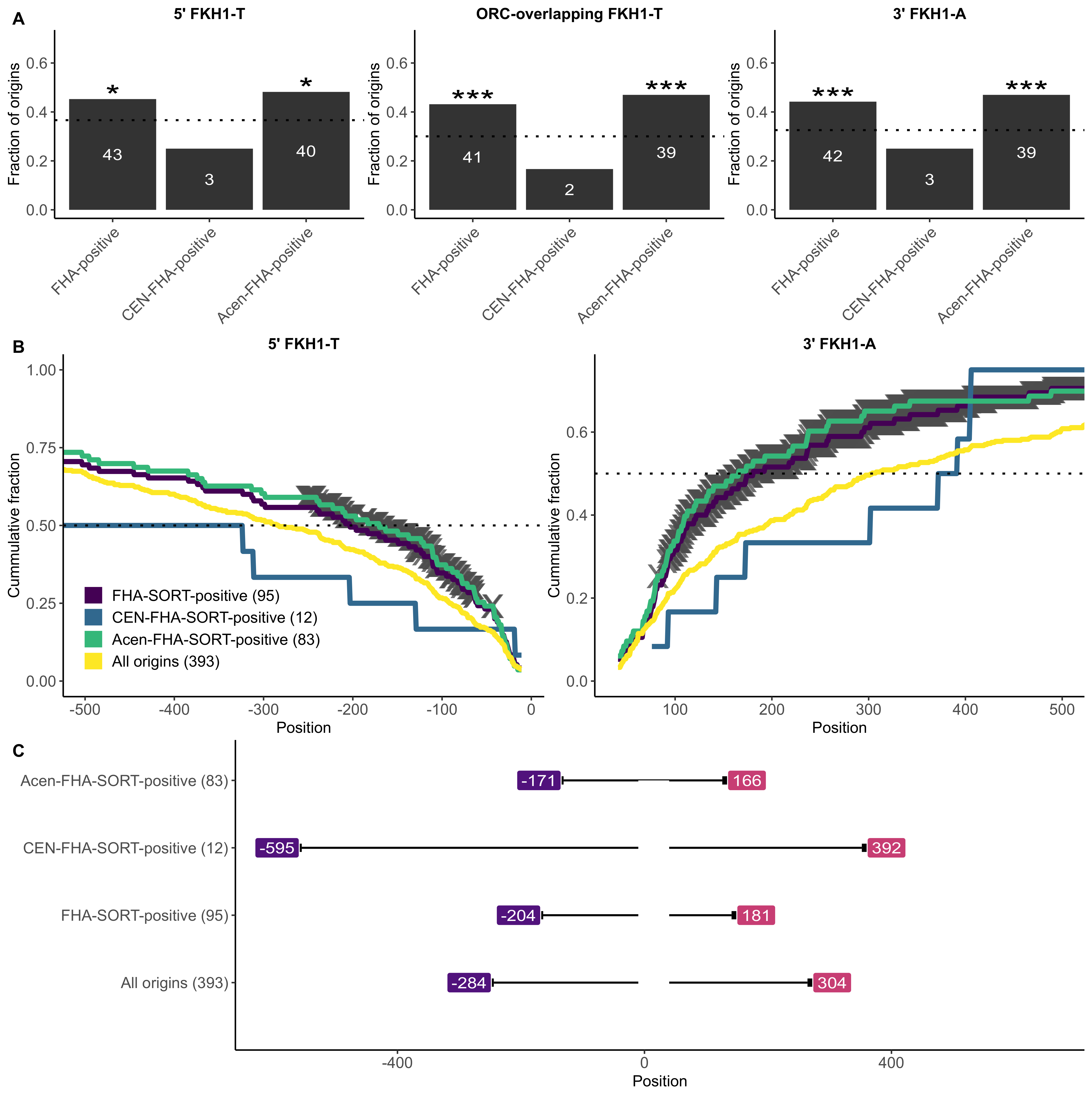

Supplement: S5 Fig — The analyses in Fig 4, main text, were applied to Cen-associated origins that were FHA positive. (A) The fraction (y-axis) of origins within the indicated group (x-axis) containing at least one match to the indicated motif within the indicated origin regions for the relevant FHA-regulated origin groups classified by SortSeq. “5’ FKH1-T” queried nucleotides -150 through -11 for FKH1-T motif matches, while “3’ FKH1-A” queried nucleotides +41 through +150 for FKH1-A matches. The “ORC-overlapping” region encompassed nucleotides -10 through +40 and was queried for FKH1-T motifs. The horizontal line indicates the fraction of all confirmed origins (n = 393) that contained a match to the queried FKH motif in the origin region under assessment. The enrichment or depletion of a given motif in any given origin group was challenged against the fraction of that motif in all confirmed origins using the hypergeometric distribution function. Significant P-values are denoted by asterisks (*, P < 0.05; **, P < 0.001; ***, P < 0.0001). In these analyses, 150 bp regions 5’ and 3’ of the ORC site were queried. (B) The cumulative fraction of origins (y-axis) in the indicated groups containing a 5’FKH-T (left) or a 3’FKH-A (right) after traversing the indicated number of nucleotides from the ORC site (x-axis). Nucleotide positions that reached P-value significance values of 0.01 are indicated by gray cross marks derived from hypergeometric distributions where at each position, the fraction of origins in the queried collection that contained a match by that nucleotide position was reached is compared to the fraction of all confirmed origins (n = 393) that contained a match by the same position. (C) Summary of the 50% accumulation point for the analyses in (B). (TIFF) [file pgen.1011366.s005.tiff]

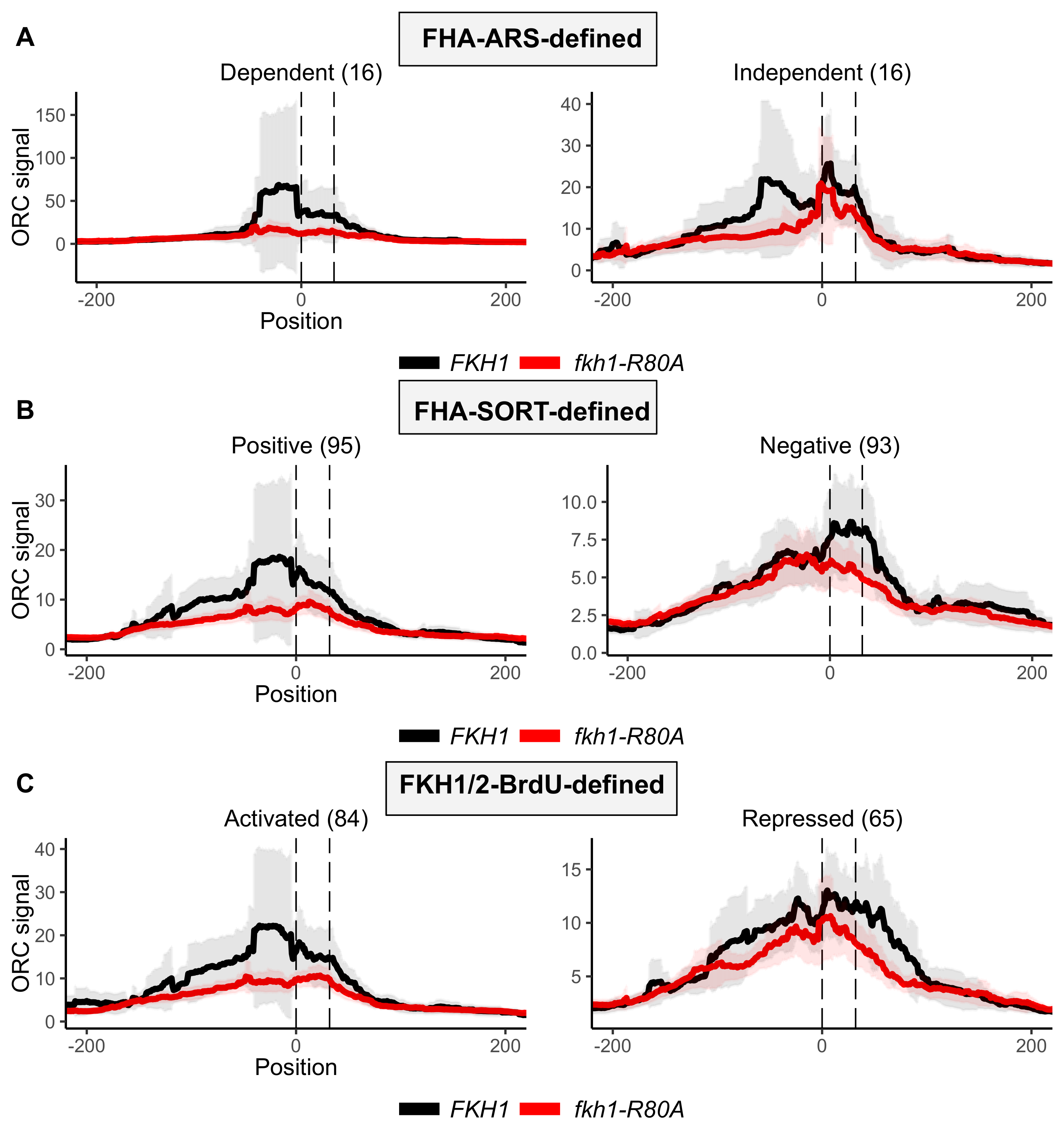

Supplement: S6 Fig — Data are as presented in Fig 5A, 5D and 5G, but with the inclusion of 95% confidence intervals indicating level of variation within the group being graphed. (TIFF) [file pgen.1011366.s006.tiff]

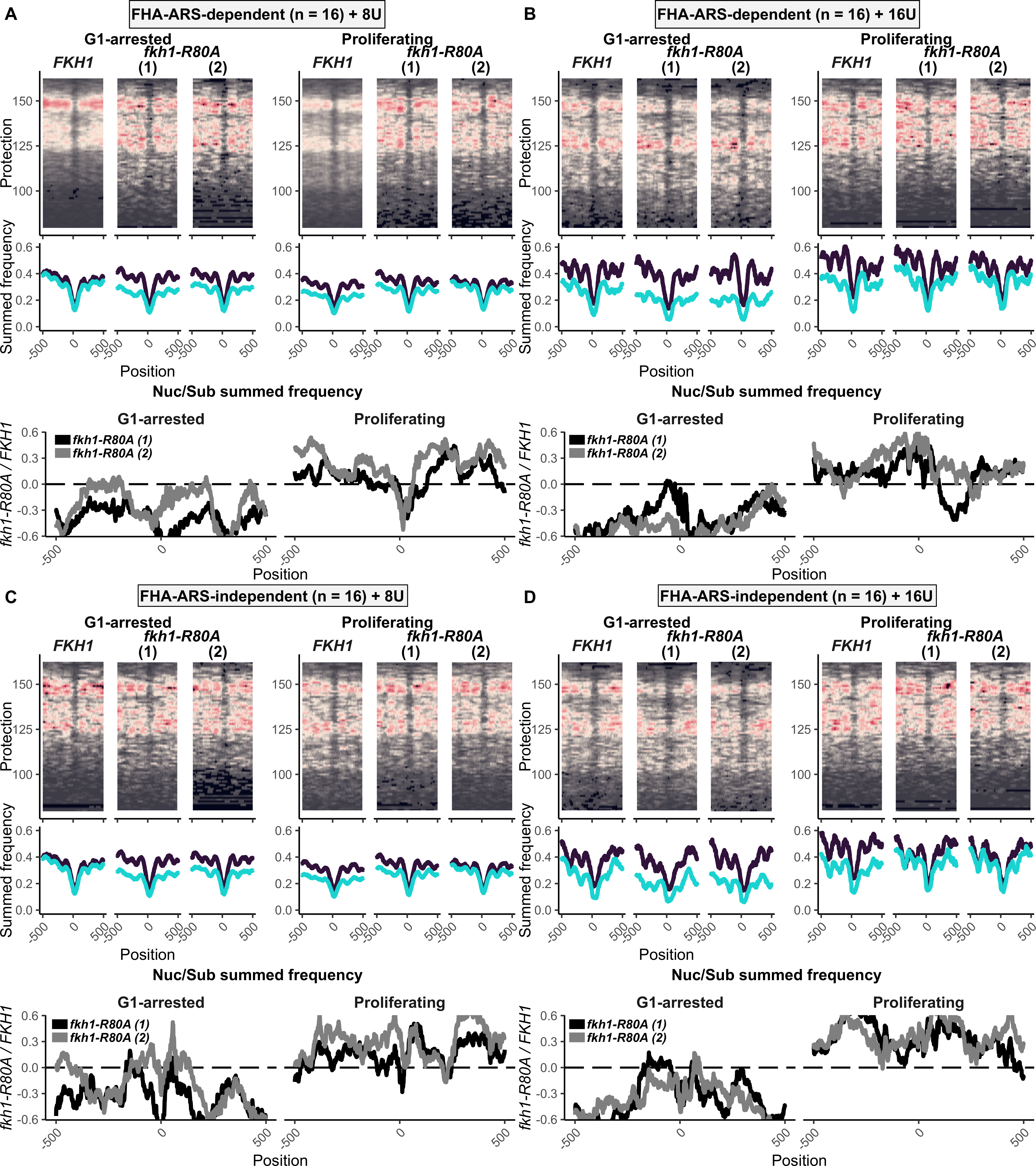

Supplement: S7 Fig — The analysis of MNaseSeq data for all origins described in Fig 6 were applied to the contrasting groups of FHA-ARS-regulated origins as defined in Fig 2A. (TIFF) [file pgen.1011366.s007.tiff]

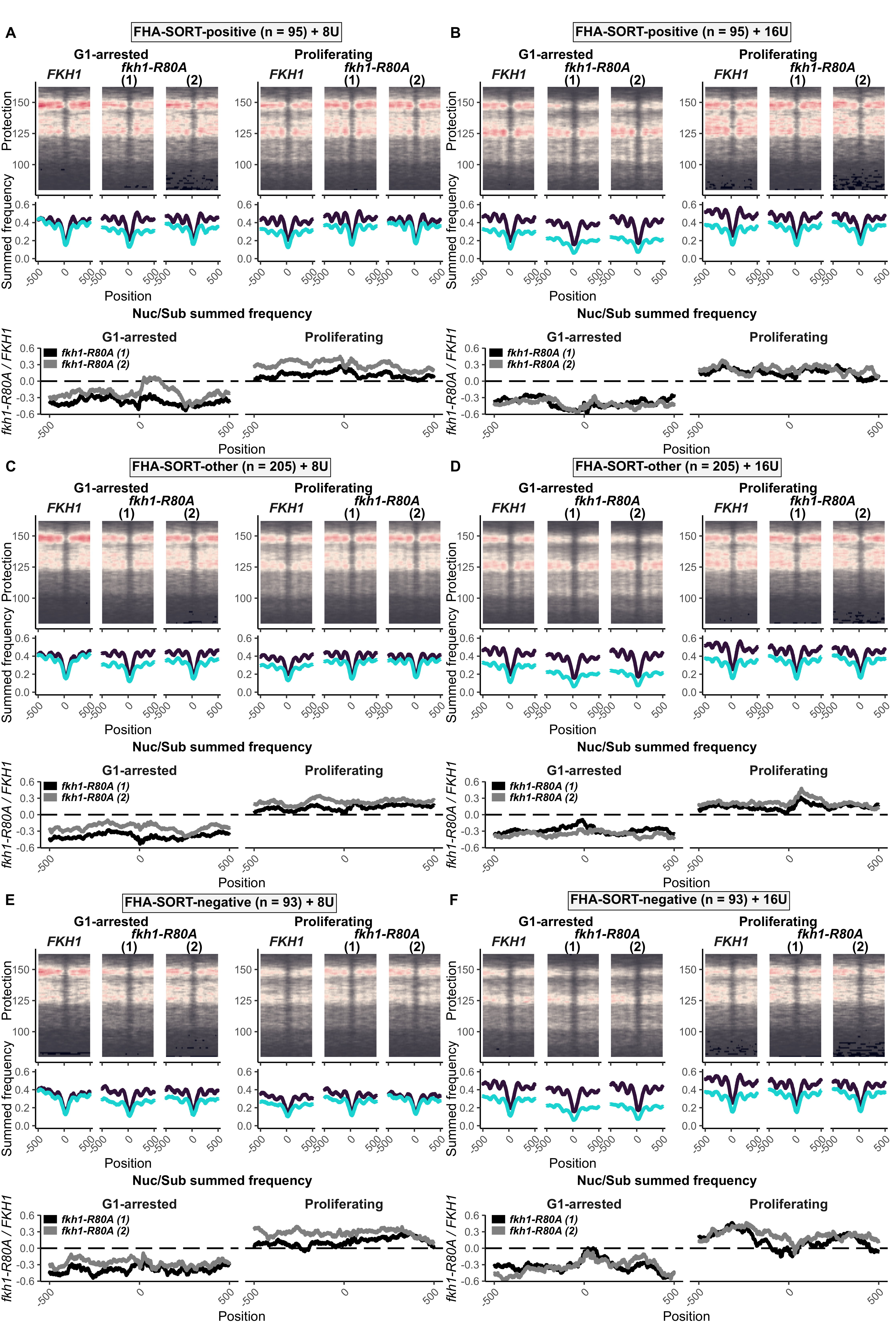

Supplement: S8 Fig — The analyses of MNaseSeq data for all origins described in Fig 6 were applied to the contrasting groups of FHA-SORT-regulated origins as defined in Figs 1 and 2B. (TIFF) [file pgen.1011366.s008.tiff]

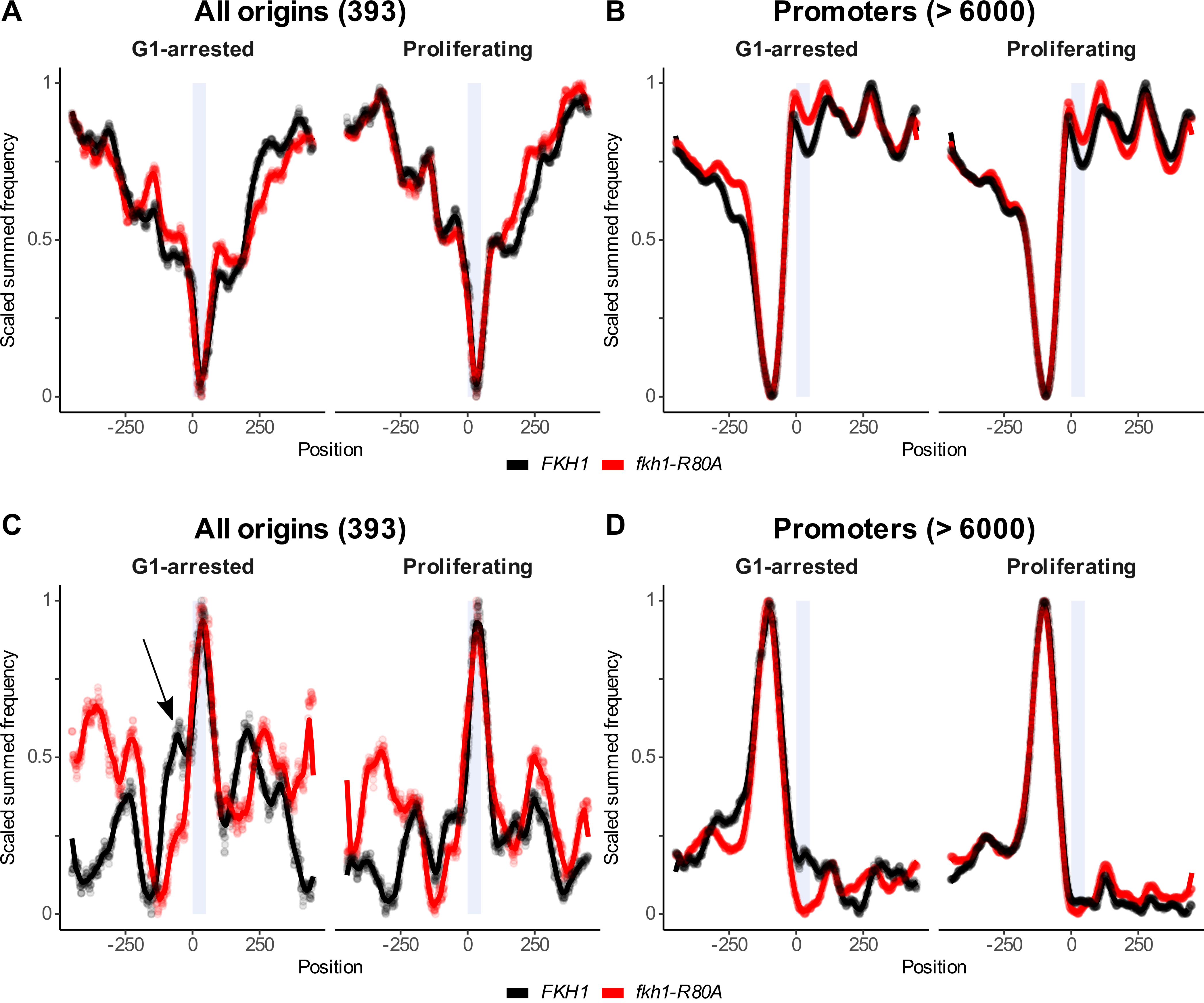

Supplement: S9 Fig — Nucleotide-resolution, scaled nucleosome (A, B) or ORC (C, D) signals for all 393 origins or all 6000 promoters in FKH1 and fkh1-R80A cells under G1-arrested or Proliferating conditions, as indicated. The arrow in panel C indicates a G1-arrest specific and Fkh1-FHA-dependent prominent 5’shoulder on the ORC-signal peak. (TIFF) [file pgen.1011366.s009.tiff]

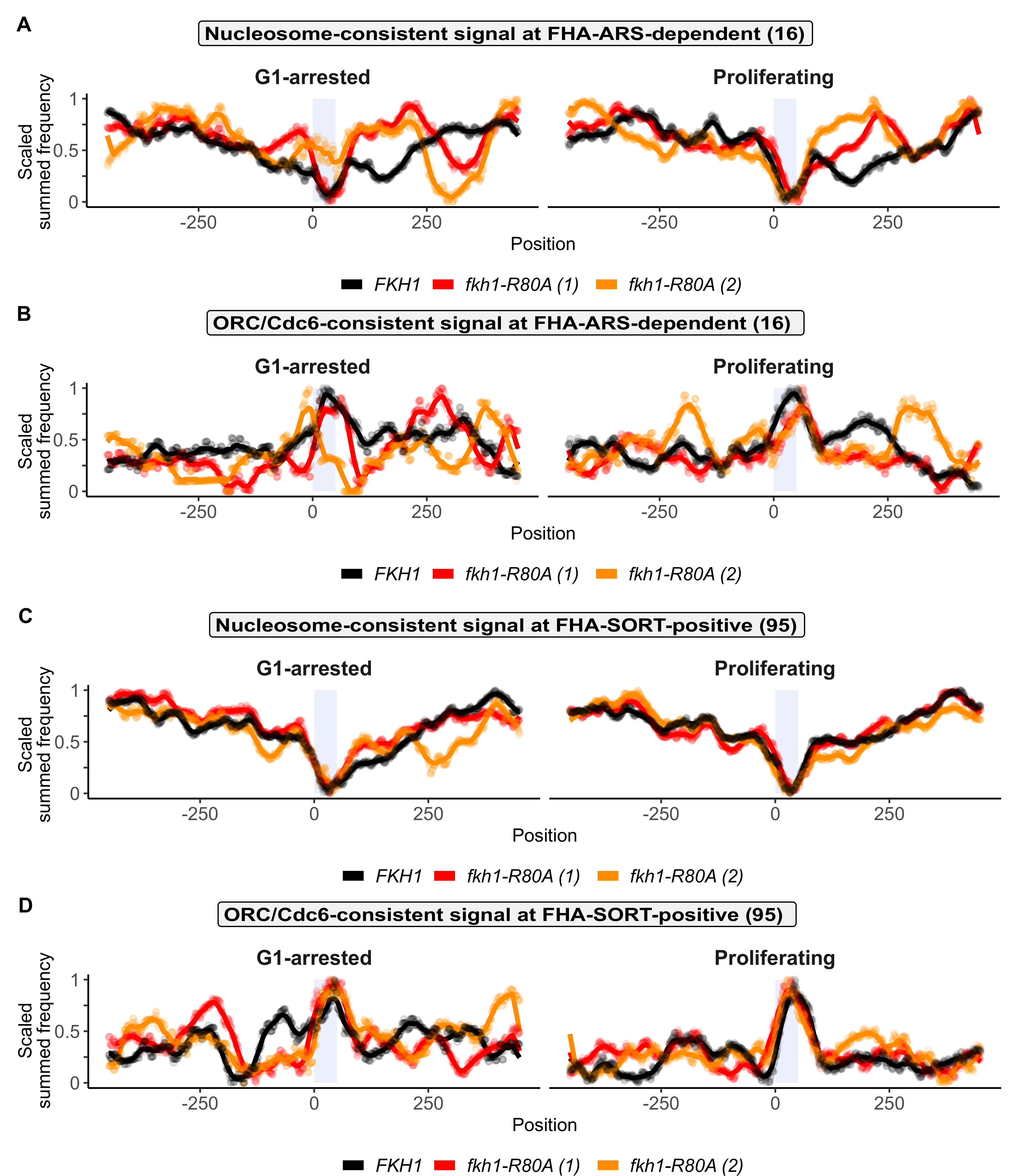

Supplement: S10 Fig — Scaled single-nucleotide signals generated from the 8 U MNase experiment for FKH1 and two independent fkh1-R80A mutant isolates for (A) FHA-ARS-dependent cohort nucleosome signals (B) FHA-ARS-dependent cohort ORC signals (C) FHA-SORT-Positive cohort nucleosome signals (D) FHA-SORT-Positive cohort ORC signals. (TIFF) [file pgen.1011366.s010.tiff]
