# Supplementary material for: The budding yeast Fkh1 Forkhead associated (FHA) domain promotes a G1-chromatin state and the activity of chromosomal DNA replication origins
Source: PLoS Genet. 2024 Aug 5;20(8):e1011366. doi: 10.1371/journal.pgen.1011366 (PMC11326605; doi:10.1371/journal.pgen.1011366)
Supplement: Attachment — Submitted filename: 2024_06_07_Response_to_Reviews.docx [file pgen.1011366.s011.docx]

**Responses to reviewers: The budding yeast Fkh1 Forkhead associated (FHA) domain promoted a G1-chromatin state and the activity of chromosomal DNA replication origins**

Timothy Hoggard^1^, Erika Chacin^2^, Allison J. Hollatz^1,3^, Christoph F. Kurat^2^, and Catherine A. Fox^1,3,4^

^1^Department of Biomolecular Chemistry, School of Medicine and Public Health, University of Wisconsin, Madison ^2^Biomedical Center Munich (BMC), Division of Molecular Biology, Faculty of Medicine, Ludwig-Maximilians-Universität in Munich, Martinsried, Germany ^3^Integrated Program in Biochemistry, University of Wisconsin, Madison ^4^Corresponding author: [cfox@wisc.edu](mailto:cfox@wisc.edu)

**Responses to Reviewer #1:**

Thank you for your comments and questions.

**Rev #1 Comment #1.**

*a. Why different replicates were used for FKH1 (n = 3, black) and fkh1-R80A (n = 2, red)?; b. Did the authors confirm their large-scale seq data by qPCR at representative origins (i.e., peri-CEN ones) or other different approaches?*

a. Biological replicates are standard practice. Here, they were particularly important because we observed variation even between independent wild-type replicates. Thus, we wanted to be confident that differences between *FKH1* and *fkh1-R80A* replication behavior in SortSeq were indeed due to these defined genetic differences rather than resulting from “noise” in the experiments due to other uncontrolled factors.

b. We used ddPCR to quantify S-phase replication phenotypes of selected origins within the previously defined FHA-ARS-dependent and -independent groups in our previously published study [[1]](https://paperpile.com/c/0ehuyH/SgNo). These ddPCR data largely confirmed (or, more accurately, predicted) the S-phase SortSeq data for these origins that we present in these studies (Please see Figure S3). We have not examined the Cen-associated origin effects at single-origin resolution by ddPCR.

***Rev #1 Comment #2*** *Fig3A. the authors compared FHA-SORT-positive based on fkh1-R80A with Fkh1/2-activated oris. Fkh2 also contains a FHA domain and plays a redundant role in origin activation with Fkh1. Is Fkh2-FHA also involved as Fkh1-FHA? What’s the effect of double FHA mutants? The relatively lower (25/93) negative implicates that it may be worth to examine double FHA mutants, although the reader agree about the dominant negative effect of R80A.*

For the purposes of SortSeq, at least for us, the *fkh2-R87A* cells grew too slowly to be practical for collecting a sufficient number of S-phase cells. We suspect that this severe growth phenotype (much more drastic than that caused by a *fkh2Δ* mutation, presumably because the *fkh2Δ* null allows Fkh1 to substitute for Fkh2) was because the Fkh2 protein is the major transcriptional regulator of “*CLB2*-cluster” genes and uses its FHA domain to recruit Ndd1 for cell-cycle restricted transcription activation of these genes [[2,3]](https://paperpile.com/c/0ehuyH/5hA4+mJ69).

**Rev #1 Comment #3**. *Fig3D. The most interesting result is 12 cen-associated origins are affected in R80A but not in fkh1/2 mutants. These CEN-ori have Fkh1 ChIP signals. Have they tried to delete FKH motifs from one or two CEN-ori as their previous work to validate this important result? Are the other 5 CEN-ori showing FHA-SORT-neg due to lacking FKH motifs? If so, inserting proper FKH motifs to one of the 5 CEN-ori (FHA-SORT-neg) to see if it is sufficient to make it FHA-dependent.*

We agree that the defect observed at Cen-associated origins in the *fkh1-R80A* mutant was an interesting result, in part because the current view is that Cen-associated origins are not regulated by a FKH-independent mechanism [[4]](https://paperpile.com/c/0ehuyH/H9LQ). However, these origins were neither the focus of our study, nor our primary goal. While we are grateful for this reviewer’s enthusiasm, the effect of the *fkh1-R80A* allele on Cen-associated origins was not the “most interesting result” for us. Rather, we were struck by how broad the Fkh1-FHA domain’s role was in yeast chromosomal origin control and ORC-origin binding. We hope that other researchers in the field are stimulated by our study to investigate Cen-associated origin observations more deeply.

**Rev #1 Comment #4.** *Fig4. A related question to above, the characteristics of 5’ FKH1-T or FKH1-A motif in CEN-ori are missing in Fig4.*

At this Reviewer’s request we have now assessed the FHA-positive Cen-Oris for 5’ FKH1-T, ORC-overlapping and 3’ FKH1-A motifs in a new Supplemental Figure, Figure S5, “completing” Figure 4C analyses. We note that CEN-oris are included in the other larger categories to which they belong, as appropriate (FHA-positive, FHA-other). The small numbers of Cen-oris combined with lack of experimental dissection of these origins at the sequence level made us concerned about including these origins as a separate category in Figure 4C.

**Rev #1 Comment #5.** *Fig5-8. How does FHA-stimulated ORC-ori binding affect nucleosome configurations? An alternative possibility is that it promotes more MCM loading, which is also known for early replication in budding yeast.*

We do not know the mechanism(s) by which FHA-stimulated ORC-ori binding affects nucleosome configurations or “stability” as in Figures 6-8. However, because ORC-origin binding is required for nucleosome positioning around the NDR [[5]](https://paperpile.com/c/0ehuyH/kTeV), one possibility is that the changes in nucleosome configurations that we *do* observe are an “indirect” byproduct of changes in ORC-origin binding dynamics. In fact, that idea was a motivation for performing MNaseSeq in *fkh1-R80A* mutants.

Our primary motivation for the MNaseSeq experiments was to test whether the Fkh1-FHA domain would be important for the characteristic nucleosome positioning around origins, at least the subset we described in our previous study as this subset required the Fkh1-FHA domain for ORC binding [[1]](https://paperpile.com/c/0ehuyH/SgNo). However, we did not observe major alterations in the origin-adjacent ordered nucleosome array (with the possible exception of the FHA-ARS-dependent group in Figure 7A, which was interesting). Because we observed a relative reduction in ORC-origin binding as measured by ORC ChIPSeq at the much larger collection of FHA-SORT-positive origins, yet saw no major changes in nucleosome positioning, our initial hypothesis was disproved. For now, we can only speculate that, while the loss of Fkh1-FHA domain function is sufficient to reduce ORC-origin binding at many FHA-SORT-positive origins, a sufficient level of ORC must remain even in a *fkh1-R80A* mutants to maintain ORC-dependent nucleosome positioning at origins.

We agree with the reviewer that the expectation is that ORC-origin binding defects result in MCM loading defects, but based on the most recent literature on this topic, defects in MCM loading need not be invoked to explain defects in ORC’s role in promoting origin-adjacent nucleosome positioning [[5]](https://paperpile.com/c/0ehuyH/kTeV).

**Rev #1 Comment #6.** *As already mentioned in their Discussion, for a deeper understanding of Fkh in origin activation, it will be of great interest to use the genome-wide approaches in this study to compare with other separation-of-function mutants, e.g., dimerization or DDK interacting, particularly combining these mutations with FHA in future.*

We agree and hope that we have an opportunity to contribute to some of these exciting experiments.

**Responses to Reviewer #2:**.

Thank you for your critique and suggestions.

**Rev #2 Comment #1.** *I worry that the chromatin data might be over-digested and may not be preserving the ORC footprint at the origins. It would be nice to show an ethidium bromide stained gel of the nucleosome ladders used for the analysis to facilitate comparison and explain the differences in the patterns with prior studies. For example, the MacAlpine group typically performs a limited-digest with MNase such that 4-5 rungs of the nucleosomal ladder are resolvable on an ethidium bromide stained gel to preserve ORC and TF footprints.*

This concern is reasonable, and we hope to improve on using MNaseSeq to map ORC-origin and other protein-DNA interactions at origins in future experiments. Upon considering the critiques and concerns of both Revs 2 and 3 about the caveats associated with relying on scaled data, we have removed the overlay panels present in the original Figures 7 & 8, as this presentation suggests an occupancy relationship, and the MNaseSeq data cannot address occupancy, only the positions where the relevant fragments are dominant. We have also modified the relevant text substantially to explain our reasoning, including a discussion of additional controls in a revised Figure S9. Finally, we have confined analyses of ORC signals to those signals that coincide with the ORC site, or dominant signals that are contiguous with those signals over the ORC site.

We acknowledge that we did ***not*** optimize our MNase titrations for trapping small footprints caused by factors such as ORC or TFs. As stated for Rev 1, our primary goal was to map nucleosome positioning. We performed duplicate experiments at two different MNase concentrations, and the 8 U digestion generated the ideal ratio of di- and mono-nucleosomes (80:20) that the Kurat lab has established as optimal for mapping nucleosome positioning. With the exception of the FHA-ARS-dependent origin group, we observed relatively minor effects of the *fkh1-R80A* allele on nucleosome positioning. All of these data are worth reporting for this important regulator of origin activity. However, because of this outcome, and because we had orthogonal data (both published and in this report, see below) about the role of the Fkh1-FHA domain in ORC-origin binding, we decided to dig further into the data to examine ORC/ORC-Cdc6-consistent fragments (80 ± 10 bp) to see if they might reveal differences between relevant origin collections (e.g. FHA-ARS-dependent, FHA-ARS-independent and so forth) in either *FKH1* or *fkh1-R80A* cells.

There are four observations that we relied on to support our interpretation of the normalized ORC signals as a reflection of ORC behavior: 1. Analyses of scaled signals in the relevant size range (80 ± 10 bp) produced an extended signal (5’ shoulder) at origins but not at promoters (While ORC obviously does not bind promoters, other factors that generate protection in this size range do [[6]](https://paperpile.com/c/0ehuyH/22Wh). These analyses are presented in a revised Figure S9. 2. We also observed a “5’ ORC shoulder” or “second ORC peak” at the same collections of origins (generally early origins, positively regulated by Fkh1-FHA) in our independent ORC ChIPSeq data in Figure 5. Thus, an orthogonal method directly dependent on the ORC protein produced a similar 5’ signal shift as our MNaseSeq analyses. 3. For the MNaseSeq data, this 5’ shoulder (extended ORC signal) was observed in G1-arrested but not proliferating cells, exhibiting similar behavior to the Cdc6-dependent extended ORC footprints observed in G1-phase in the MacAlpine study [[7]](https://paperpile.com/c/0ehuyH/EkbL) and also consistent with biochemical and structural data on ORC-Cdc6 (e.g [[8,9]](https://paperpile.com/c/0ehuyH/8imJ+wRzM)). 4. Both the 5’ ORC ChIPSeq signals in Figure 5 and the 5’ ORC signal shoulder in the MNaseSeq analyses were lost in *fkh1-R80A* mutants. It is also important to note that MNaseSeq experiments do not exist in a vacuum. Instead, their value is our ability to interpret them in the context of other independent data. The key point for this study is that the Fkh1-FHA domain was necessary for the G1-arrested extended ORC-consistent signals at origins over the ORC site at FHA-SORT-positive origins, while having only minor (or opposing) effects on putative ORC signals over the ORC site at FHA-SORT-negative origins.

**Revised Figure S9**

**Rev #2 Comment #2.** *The generation of subnucleosomal complexes from (presumably over-digestion) was very interesting and suggest that there might be differential nucleosome accessibility/breathing at origins that is FHA dependent. This may be an opportunity for a more focused analysis on the distribution of nucleosomes and different subnucleosome complexes (not just in aggregate), but a breakdown of the individual species for each flanking nucleosome. I think there might be some insightful patterns that were missed in the bulk analysis captured by the teal and black boxes.*

This point is well taken, and a very important goal for the future is to extend these analyses to include subnucleosomal particles as well as to generate data that allows us to examine individual loci, and mutant loci, with greater confidence. For our lab a priority is to generate additional experimental data (e.g. at lower MNase concentrations, as suggested by this reviewer) before jumping into deeper analyses of these existing data. We would also like orthogonal data *in vivo* that leads to similar conclusions about “nucleosome stability” before investing in more MNaseSeq data.

**Rev #2 Comment #3.** *ORC footprints were almost non-existent in the raw chromatin data prior to "positional probability" normalization as described in (Figure S8). I just don't see how the authors got from the middle panel to the third panel as there is a negative inflection in the ORC summation data in the first panel that somehow becomes a peak in the third panel.*

We apologize for the confusion and frustration it might have caused. We have made a number of changes to the text, supplemental figures and main text figures to clarify issues relevant to these concerns and to be more circumspect in our interpretations. Some of these points were discussed above in the response to Rev #2 Comment #1. Additional changes noted here include: 1. Relabeling the y-axis to “Scaled summed frequency” to more intentionally convey that these are normalized signals. Because the ORC-consistent fragments make up a much larger fraction of fragments that can be detected over the origin/NDR region, the scaled ORC-signal is obviously going to be highest at this location. Nevertheless, as discussed above, several observations supported the inference that these “ORC-signals” reflected ORC behavior and were not merely “noise”, at least at FHA-SORT-positive origins. 2. The analyses of FHA-SORT-positive origins in Figure 8B indicates that ORC signal peaks do not merely correspond to regions with severely depleted nucleosome signals. Specifically, the prominent 5’ shoulder on the ORC peak around position -100 is comparable in intensity to the main ORC peak over the ORC site, yet it corresponds to a region with a relatively high nucleosome signal. This signal is also only observed in the G1-arrested condition and is Fkh1-FHA-dependent, even as the nucleosome signals are unaffected by these same conditions (Figure 8E and Figure 8D).

**Rev #2 Comment #4.** *I struggled with Figures 7 and 8 and I am concerned that some of the ORC specific differences might be driven by an outlier or two especially where the number of origins in the set was limited. Is it possible to put confidence intervals on the curves? Or present the positional probability data as heatmap (with each row representing an origin)?*

Outliers, especially when analyzing small groups, is a general concern with any type of genome analyses, and holds for the nucleosome as well as “ORC specific” signal differences we observe. This concern is larger for Figure 7 because of how small the FHA-ARS-dependent origin group is (n = 16). To clarify one point, however, for this small group, we did not emphasize the “ORC-specific” differences between FHA-ARS-dependent and FHA-ARS-independent origins, as those were evident but not the dominating FHA-dependent differences between these two groups. Rather, we emphasized the nucleosome differences, which were the dominant “difference” for FHA-ARS-dependent origins in G1-arrested vs proliferating conditions, between *FKH1* and *fkh1-R80A* cells, and between FHA-ARS-dependent versus FHA-ARS-independent origins. The one advantage of this small group is that we have solid experimental data about the requirement of the highly enriched 5’ FKH-T site [[1]](https://paperpile.com/c/0ehuyH/SgNo). Finally, even taking into account this reviewer’s valid critique about these data, there is complementary and consistent data from other experiments. In particular, the G1-specific FHA-dependent depletion of nucleosome signals were on the 5’ side of the ORC site at FHA-ARS-dependent origins, near the experimentally validated 5’ FKH-T sites. While this observation is not definitive—many more experiments are required to test whether there is a causal connection between 5’ FKH-T sites and these nucleosome alterations—it is nevertheless an intriguing result worth reporting. However, we fully acknowledge that this result is only an intriguing correlative outcome at this point, and stress that point in the discussion.

There is reduced concern that a small number of outliers affect the FHA-SORT-positive origin group because this group has ≈ 100 origins. Specifically, while it is implausible that all origins within this group behave identically, it is likely that substantial subsets behaving similarly contribute to the mean behavior that is reported. In contrast to the FHA-ARS-dependent group, for the FHA-SORT-positive, the G1-specific ORC signals *were* the more striking difference that was emphasized.

We generated a new supplementary Figure, **Figure S10** to address some concerns about the robustness of these nucleosome and ORC signals. Specifically, MNAseSeq experiments were done with two independent *fkh1-R80A* replicates, *fkh1-R80A-1* and *fkh1-R80A-2*, the same two independent replicates that were used for SortSeq. A challenge was that these two replicates were digested to different extents at the 8U MNase experiments, with the *fkh1-R80A-1* replicate showing greater digestion than the *fkh1-R80A-2* replicate (see Figure 6) . In the main text, we used *fkh1-R80A-1*, which was digested to a slightly greater extent, for defining ORC and MNase signals in Figures 7 and 8, because smaller DNA fragments were more abundant, and this replicate gave the more pronounced SortSeq phenotype. In Figure **S10**, we show analyses of both replicates for both groups of FHA-positively regulated origins FHA-ARS-dependent (Figure 7) and FHA-SORT-Positive (Figure 8). Not surprisingly, and consistent with Rev #2’s concerns, for the small FHA-ARS-dependent group (n=16), we observe substantial variation between the two replicates for the nucleosome and the ORC signals, likely reflecting the different levels of digestions combined with the small number of origins in this group. Nevertheless, for the nucleosome signals, which were the major chromatin feature that was G1-arrest specific and Fkh1-FHA-dependent that was emphasized for this group, the enhancement in nucleosome signals observed in *fkh1-R80A* cells is observed for both replicates. Thus our conclusion that the Fkh1-FHA domain promotes the NDR at these origins holds for two replicates. More strikingly, for the larger group of FHA-SORT-positive origins, the overlap of the two independent *fkh1-R80A* replicates is robust for both nucleosome- and ORC-signals. For this group, the major signal difference emphasized *was* for ORC signals and, notably both independent *fkh1-R80A* replicates generated the same effect on the extended G1-ORC signal peak—specifically, the 5’ extension was lost. These extra analyses support the conclusion that these G1-specific differences in ORC-signals were not simply “noise” but rather related to the *fkh1-R80A* allele. Whether they are due to ORC binding and DNA protection *per se*, the concern of this reviewer as we interpret it, or instead some indirect read-out of ORC behavior or other protein-remodeling events at these origins in G1, will require further investigation. Nevertheless, as stated above, a number of orthogonal observations support the idea that these signals reflect ORC behavior (i.e. a preponderance of evidence—these data do not stand alone, but are interpreted in the context of other data, generated by both us and other labs). In addition, what these data clearly demonstrate is that there is a G1-arrest specific Fkh1-FHA-dependent change in origin architecture at FHA-SORT-Positive origins that can be detected for small fragments even when no changes in nucleosome behavior can be detected. The $40 million dollar question is the identity of the Fkh1-FHA protein partner(s) responsible!

**New Figure S10**

**Rev #2 Comment #5.** *The upstream 'double ORC' peak that is in FHA positive sort and Fkh1 dependent (Figure 8B) -- could the upstream peak represent a Fkh1 footprint?*

To clarify, we observed the “double peak” (what we called a 5’ shoulder on the ORC peak) for ORC-consistent signals for FHA-SORT-Positive origins (n = 95), but only a minor 5’ shoulder on the ORC peak due to a 5’ extension for the smaller group (n = 16) FHA-ARS-dependent origins. For both groups, however, these “extended” ORC-consistent footprints were observed in G1-arrested but not proliferating cells. Because MNase footprinting in vivo is “factor agnostic”, this extension of the ORC-consistent footprint could conceivably represent ORC in a complex with Fkh1; the data cannot address this issue. All we can conclude is that the G1-arrested ORC-consistent footprints change substantially in response to loss of the Fkh1-FHA domain, as defined by the *fkh1-R80A* allele, and that the change is in the direction of much tighter, narrower signal that appears much more similar to the ORC-consistent signals observed in proliferating cells.

As stated above, we are not interpreting these data in a vacuum, but rather in the context of other published data, including the MacAlpine study referenced above but also biochemical and structural work from other labs, in particular the work of Christian Speck and Bruce Stillman. Based on all these studies and the current thinking, the most parsimonious interpretation is that an ORC-Cdc6 complex likely underlies the G1-specific extended ORC signals. It could be that Fkh1-FHA stimulates ORC stability at origins in G1 by recruiting Cdc6, as the ORC-Cdc6 complex is more stably associated with origin DNA than ORC alone. However, there is no evidence yet for a direct Fkh1-FHA-dependent interaction with Cdc6, and we have not been able to obtain any evidence for a direct Fkh1-FHA-dependent interaction with any ORC subunit. Alternatively, these signal changes could be in response to the recruitment of the DDK and other pre-initiation (early MCM activation) factors, as there is evidence that these are recruited to early origins prior to the onset of S-phase (e.g. [[10]](https://paperpile.com/c/0ehuyH/2An3)).

**Responses to Reviewer #3:**

Reviewer #3 had several technical concerns about the study, with major concerns being related to the “noise” or “variation” present in our S-phase SortSeq data that caused them to worry about the validity of our downstream conclusions from computational analyses. Reviewer #3 was also troubled by our over-enthusiastic interpretation of this variation being “*biologically relevant to stochastic control*”, a fair criticism that forced us to think more critically. We have done our best to respond to these concerns as comprehensively and thoughtfully as possible. We believe that this reviewer’s concerns, and editorial suggestions with respect to them, have helped us to strengthen the study. Finally because of this reviewer’s concerns, we include additional data in Figures included with this response, as below.

**Rev #3 Comment #1.** *As shown in Figure 1 and discussed in the text, the Sort-seq data are noisy and relatively inconsistent between replicates.*

The reason the SortSeq data appear “noisy” is because we have plotted the data in a way that the variation between our independent experiments (independent *FKH1* and *fkh1-R80A* haploid isolates from a backcross, treated completely independently, with the resulting data processed completely independently (following Batrakou [[11]](https://paperpile.com/c/0ehuyH/h179))) is explicit. To the best of our knowledge, our study is the first to have performed independent biological replicate experiments of SortSeq data in this way, and presented the data generated from them in such a way that the variation between biological replicates is evident in a single Figure (Conrad Nieduszynski shared this point to us at a conference in a personal conversation.) Therefore, we are unaware of any published experimental baseline for the “noise” one would expect from these types of experiments, even for a wild-type W303 strain.

Because we observed some variation between WT replicates, the salient issue for us was whether we could conclude that there were effects on origin usage that could be attributed to the *fkh1-R80A* allele as opposed to being the result of noise in the experimental system (due to biological and technical variables that we were unaware and/or were not controlled for).

While we observed variability between outcomes even *within* a given genotype (either *FKH1* or *fkh1-R80A*), this variation was **not** sufficient to explain the effect that the *fkh1-R80A* mutant allele had on many discrete regions of the genome, including many origins (i.e. FHA-SORT-positive), the loci of this study’s focus. For example, in Figure 1A, the major focus of this reviewer’s critique, the *fkh1-R80A* strains generate reductions in the peaks corresponding to several efficient origins (downward arrows), while increasing the signals associated with several minor peaks corresponding with later replicating regions/less efficient origins (upward arrows). For many of these origins and others throughout the genome, the *fkh1-R80A*/*FKH1* signal differences were larger than the variation or “noise” (95% confidence level) observed *within* the same genotypes. Thus, we concluded that the *fkh1-R80A* mutation affects the activity of many origins.

We have rewritten the Results section to emphasize these points, as well as added additional analyses to Figure 1 (see **revised Figure 1B**) as requested by the editor. In discussions with the editor, part of the “noise” issue may arise from higher resolution analyses we performed relative to other studies. Specifically, we examined the data in 1 kb increments and performed minimal smoothing. Scientists disagree about how much smoothing is appropriate for a given experimental approach, and we may have erred on the side of “not enough” smoothing, making the data difficult to view. Nevertheless, as shown in the included in Figure S1 and Figures for Reviewers (**RFig1-RFig4**, below) we actually observed fairly consistent results across replicates, with the most extensive variation being confined to particular regions of the genome.

**New Figure 1B**

**Revised Figure S1**

**Revised Figure S2**

**Rev #3 Comment #2.** *The authors suggest in the text that stochastic origin use likely underlies this variability, but Sort-seq analyses generally uses millions of cells (30M in the original description of the method by the Nieduszynski lab, down to ~1M for the lowest-input analysis of which I am aware).* ***Across such a large number of cells, variability due to stochastic origin use should smooth out to give much more reproducible profiles.*** *A more likely scenario in my opinion is variability in gating, such that cells in early or late S-phase are under- or over-represented. It is very difficult to glean meaningful information about gating from the low-resolution data provided in Figure S1.*

We have now replaced the smoother cell-cycle graphs with the actual histograms and gating cut-offs for each independent experiment that used the Sony MA900 Sorter in revised Figure S1. As can be seen from these raw histogram data, the independent proliferating populations behaved quite homogeneously, and our gating was as stable as experimentally possible.

Two different but related concerns are raised by this comment: 1. Whether the stochastic control of origin use can be detected in SortSeq (ensemble) experiments that result from an analyses of millions of individual S-phases and 2, Whether differences in gating S-phase cells between sorting experiments are sufficient to explain the noise/variation we observe within independent experimental replicates of the same genotype.

Like this reviewer, we also expected less variation between replicates of the same genotype than we observed for our SortSeq experiments, and for the same reason. In a discussion with the editor, we came to the conclusion that our higher-resolution, less smoothed data may be contributing to the appearance of more variation than is biologically meaningful. Nevertheless, we were concerned, given that we had the capacity to generate higher-resolution data, with potentially “over-smoothing” the data. In addition, Conrad Nieduszynski has reported that the stochastic nature of termination zones can be deciphered from ensemble experiments, but we admit that the math to explain why is beyond our expertise [[12]](https://paperpile.com/c/0ehuyH/5hdU).

However, this reviewer is 100% correct that slight variations in gating could explain some of the variability we have observed between isogenic replicates (please see revised Figure S1), and we have now included that possibility in the rewritten text relevant to Figure 1. Other issues that could contribute to variation between biological replicates include slight differences in the growth conditions for independent cultures (slight differences in temperature or media), slight differences in library construction and/or the actual sequencing itself. However, in our opinion, these possibilities only underscore the importance of performing replicates when examining mutants that alter origin activity/distribution. Regardless, as stated above, even with the noise that is present in these data, it is clear that the *fkh1-R80A* allele had an effect on origin activity across yeast chromosomes, as many regions are altered beyond the level that can be statistically attributed to noise.

We have modified the Results and Discussion sections to downplay our overzealous embrace of the potential for stochastic control to cause the variation that we observed. However, we still posit that some biological differences that are not typically controlled for could account for some of this variation. For example, our practice is to examine isogenic replicates (*FKH1* and *fkh1-R80A*) obtained from a backcross (W303 background). Thus, it is possible that meiotic independents possess undefined polymorphisms that contribute to variable outcomes between two supposedly genetically identical haploids. We have not sequenced deeply enough to map such polymorphisms. In addition, both independent meiotic and mitotic haploids can possess distinct rDNA copy numbers that are stable over millions of mitotic divisions [[13]](https://paperpile.com/c/0ehuyH/5YsR). Effects of rDNA copy number on the activity of non-rDNA origins has been reported, at least in mutant strain backgrounds, and they may occur even in wild-type cells (e.g. [[14–16]](https://paperpile.com/c/0ehuyH/3tXy+KwoC+Jr5C)). This issue requires more systematic examination. Furthermore, a recent study reveals an even greater complexity to the relationship between rDNA copy number and replication demand than already acknowledged that is both exciting and somewhat daunting to contemplate [[17]](https://paperpile.com/c/0ehuyH/lA5p). While the rDNA issue is an extremely important one, and it may be relevant to some of the variation we observe, in our view it is beyond the core goals of this study, and deserves the focused attention it is receiving from labs with the expertise to tackle this complex topic.

To respond more completely to this reviewer’s concerns we have also included additional data for the Reviewers: **1.** We include un-summarized data of the wild-type replicates in our study (**RFig1**). If these data suggest anything, it is that we likely ***underestimated*** the impact of the *fkh1-R80A* allele on origin activity because one of the included WT replicates deviated more from the others. However, this replicate did not deviate equally at all regions of the genome (see right end of chromosome II, **RFig1**), and so we were not comfortable discarding this replicate. We have since performed additional sequencing of *FKH1* replicates (**RFig2**). The outcomes are consistent.

**RFig1**

**RFig2**

For downstream computational analyses, we used a stringent cut-off for establishing our origin groups to better enhance the differences between origins on the extremes for computational comparisons (Figure 1). This step is conceptually similar to analyzing only the loci that overlap between two independently generated data sets, which is more commonly done, and should mitigate the effect of variation/noise on final outcomes. For our goals, it was important to be explicit about the variation we observed, hence we plotted mean +/- 95% confidence intervals.

**2.** An independent SortSeq experiment of one replicate each of our *fkh1-R80A* and *FKH1* strains was performed for us by Conrad Nieduszynski (n = 1 for WT (*FKH1*) and n = 1 for *fkh1-R80A*). As can be seen in **RFig3** and **RFig4,** the scans of chromosomes II and V, respectively, the major conclusions hold.

**RFig3**

**RFig4**

KDE analyses on all origins performed for our mean values (as in Figure 1 (WI, for Wisconsin) and for the single values by Conrad Nieduszynski (UK) also indicate highly similar outcomes (**RFig5**). The FHA-Positive origins defined by the data generated for this study (WI), and the data generated by Conrad Nieduszynski (UK) overlap significantly (**RFig6**).

**RFig5**

**RFig6**

**3.** As an additional layer of QC for our “technique” we have also performed SortSeq experiments on several other mutants of interest, and each of these produce outcomes quite distinct from those produced by the *fkh1-R80A* allele. As a highly relevant example, we performed SortSeq experiments on the *mph1-2TA* allele (**RFig7**). The mph1-2TA mutant protein abolishes the Fkh1-FHA-Mph1 direct protein-protein interaction (by substituting the two threonines whose phosphorylation is essential for the Fkh1-FHA-dependent interaction with Mph1 to alanines) [[18]](https://paperpile.com/c/0ehuyH/9IeQ). This interaction is required for the directionality of mating-type switching. These experiments were performed contemporaneously with our *fkh1-R80A* SortSeq experiments, and we used the same gating selections and direct monitoring approach during our SonyMA900 Sorting protocol. For two independent replicates of *mph1-2TA*, we observed virtually ***no*** variation in chromosomal scan data (**RFig.7**). In fact, the data from these two replicates showed less variation than we observed even for our five independent wild-type replicates. Whether this outcome resulted from “technical good luck” or underlies some relevant biology remains to be determined.

**RFig7**

However, these data make clear that we are not inherently incapable of producing reproducible replicates by this approach, even with the relatively high resolution display of the data that we are using. Mph1 is the only Fkh1-FHA-dependent partner that has been mapped at the level of the specific pT peptide. Therefore, our hope was that we would observe a *fkh1-R80A*-like origin phenotype, perhaps attenuated or specific for only a subset of origins. However, that was not the outcome, and thus we determined that these data did not belong in this manuscript.

In summary, slight alterations in gating could explain some of the variability between “genetically identical” biological replicates, as could aspects of biology that have not been controlled for or thoroughly examined. The text has been modified to de-emphasize the discussion of “stochastic control” underlying the variation between genetically isogenic replicates. **We stress that even taking into account the variation we observe between replicates, our primary conclusion, that the *fkh1-R80A* allele impacts origin distribution across chromosomes, is supported by the data.**

**Rev #3 Comment #3.** *There are also possible coverage issues, as evidenced by the sharp peaks at ~200kb and ~750kb in Figure 1A (similar issues are also visible in Figures 1B, 2B and 3A). Such* ***sharp transitions*** *in coverage in some but not all datasets suggest that there might be problems with the quality of input material – if this were an analysis issue one would expect similar problems to manifest across all datasets. The sequence coverage reflected in the raw data files indeed appears to be insufficient for Sort-seq (considering data loss due to rDNA, mtDNA and PCR duplicates).*

We hope the additional data we have provided in the revised text and in the Figures for Reviewers alleviates serious concerns about coverage issues. Comprehensive analyses of annotated reads indicated that each replicate achieved the minimum coverage recommended for these experiments and a summary is now included in the Methods section.

In terms of the sharp transitions (i.e. spikes) in the chromosomal scans that were particularly noted for the *fkh1-R80A* data in Figure 1A: these were caused by two features of the methodology in data processing to generate these scans: 1. Following the Batrakou processing pipeline [[11]](https://paperpile.com/c/0ehuyH/h179), outliers within the defined 1 kb bins (generated independently for each sample) were discarded. (*Note: Such outliers occur in other SortSeq data, but are not observable in chromosomal scans from a single experiment, as is typically graphed, because the discarded value is simply a blank spot that gets smoothed over*.) 2. Because we wanted to be transparent about the variation we observed between genetically identical replicates, we generated the mean value for each 1 kb bin among the replicates, and plotted that mean value and the ± 95% confidence interval for that 1 kb mean. Because we plotted the mean value between two replicates for *fkh1-R80A*, any outlier in one data set meant that the S-phase copy number value plotted for that chromosomal locus *reverted to the value of the other replicate*, causing a spike (essentially scar of an outlier in one sample) in the data, as the only value for that 1 kb bin was the value from the other replicate.

Because these spikes were distracting, and take the the reader’s focus away from the main points of the experiments, we have discarded bins that were not represented by both independent biological replicates and regenerated the chromosomal scans for the *fkh1-R80A* samples, where the spikes were quite obvious (as suggested by the editor.) There were a few spikes in the wild-type (FKH1) scans as well, but fewer, and so we did not perform the same scar-removal analyses for the wild-type scans.

**Rev #3 Comment #4.** *Based on the data deposited in the SRA, the GC content of the sequenced DNA is a long way from that expected for S. cerevisiae (e.g. SRR27983880 has 54.8% GC; SRR27983878 52.8% GC, and SRR27983879 is 53.8% GC, while SRR27983882 is a more reasonable 39.6% GC, mirroring the actual GC content of the yeast genome). I find this discrepancy concerning, and speculate that it may represent a substantial amplification bias.*

We hope that the additional data, modifications to the study, and explanations reduce concerns about the % GC content. We now make our own libraries in a laboratory that also works with prokaryotic genomes. Thus, some of our libraries may be contaminated with bacterial genomes or plasmids. We sequenced these experiments deeply enough that we obtained sufficient read coverage per chromosome (following Batrakou [[11]](https://paperpile.com/c/0ehuyH/h179)). All samples were amplified to the same extent, with the 1/10 adaptor dilution and 7 amplification cycles using the NEBNext Ultra II kit, and these points are now included in the revised Methods. We also better discuss read coverage in the revised Methods section.

**Rev #3 Comment #5.** *The points above make it hard for me to draw firm conclusions from much of the downstream analysis.*

We understand and have made efforts to rectify your major concerns, as discussed above. However, this Reviewer’s critique did seem highly focused on the parts of the data in Figure 1A that were anomalous rather than the majority of the data that produced clear and significant differences between *FKH1* and *fkh1-R80A* samples. For example, while there were a few anomalies in Figure 1A, the vast majority of the chromosomal scan shows clear effects of the *fkh1-R80A* allele that lie outside of the 95% confidence level that defines “noise.” Moreover, we assert that the downstream analyses, which focuses on large collections of origins formed based on similar behaviors defined at a selected cut-off, actually mitigates technical issues that might affect a few loci, allowing us to define general features that distinguish differentially regulated origins.

**Rev #3 Comment #6.** *Is it possible, using Sort-seq, to deconvolve origin firing efficiency vs firing time? A change in either could give rise to a similar Sort-seq profile, but these are distinct behaviors.*

Efficiency and timing are operationally defined as distinct behaviors, but they are often not distinguishable because they are each controlled by the exact same intrinsic value—the probability that an origin will act [[1]](https://paperpile.com/c/0ehuyH/SgNo). A mutation in an origin that alters its efficiency will alter its timing, and vice versa. In a bulk S-phase SortSeq experiment, like the ones reported in this study, I think the earliest replicated regions of the genome will have the highest S-phase copy numbers, and these will naturally correspond to the most efficiently replicated early origins (the highest peaks, S phase copy numbers approaching 2N.) Regions that replicate later in S-phase, whether they are origins that fire later or regions that are replicated passively, will have lower S-phase copy numbers. A later acting, efficient origin (rare, but some exist if they exist in an “origin desert”) will form a clear peak in an S-phase SortSeq experiment, but that peak will not be as high as the peak of an early, efficient replication origin peak. So, I think to some extent efficiency and timing can be deconvoluted in SortSeq data by using both qualitative (is the peak a single isolated, sharp peak?) and quantitative (S-phase copy number of peak apex) information.

We use the terms “early” and “late” based on the assignments of these origin’s Trep times in the Yabuki et al study [[19]](https://paperpile.com/c/0ehuyH/LWOK) as a convenient, familiar and widely accessed resource for clustering origins with similar replication times *in vivo*. Trep times do not refer to the time an origin locus fires, rather they refer to the time in S-phase when 50% of that origin locus has been duplicated—regardless of whether it was duplicated due to its own firing or duplicated passively by a replication fork. The biological impact of the *fkh1-R80A* allele on genome replication at all regions, not just origins, is now better emphasized in revised Figure 1B, as recommended by the editor.

**Rev #3 Comment #7.** *Some of the analysis feels over-worked from my perspective. For example, the sequencing data in Figure 4 suggest that there is a large ORC peak immediately upstream of the origin in FKH1 cells, which is lost in fkh1-R80A. The interpretation (or at least, my interpretation of the interpretation) is that there is a change in ORC positioning rather than the simpler explanation than loss of a specific peak while the rest of the region remains relatively unchanged.*

I think that the reviewer is referring to Figure 5, not Figure 4. In addition, we are unclear on how this Reviewer’s interpretation is different from ours. For the ORC ChIPSeq results in Figure 5, for positively regulated origins, the apex of the peak occurs 5’ of the ORC site. In *fkh1-R80A* cells, this apex is lost, while the signal over the ORC site itself is reduced but not lost. Based on how these experiments are performed, i.e. using formaldehyde to crosslink protein to DNA, the simplest explanation is that ORC is able to crosslink to regions 5’ of the ORC site efficiently in *FKH1* cells but not in *fkh1-R80A* cells. “Positioning” may be the wrong word to use, and we have edited the text. We have also removed the “positioning panels” C, F and G from Figure 5 as the phenomenon is clear from the ORC ChIPSeq scans and we were concerned that these panels contributed to the “over-worked” appearance of these data.

**Rev #3 Comment #8.** *On a similar theme, the analyses in Figures 7 and 8 appear to have normalized such that the minimum and maximum values within the analyzed range are 0 and 1, respectively. I suspect that this normalization has a big impact on the interpretation. Considering Figure 7F, if the effect of the FHA mutation is to essentially abolish the ORC peak, appropriately normalized data for ORC coverage would almost certainly look similar between WT and mutant outside the peak region, with the absence of a peak being the major difference. Artificially expanding the y-axis by normalizing to the maximum value in the range makes it look as though new ORC occupancy peaks appear in the mutant. As an additional note here, the data presentation in Figures 7 and 8 is so busy that it’s almost impossible to take anything away without extreme effort on the part of the reader.*

We have simplified these figures by removing the nucleosome and ORC signal profile overlay figures, which were misleading, and emphasizing the limitations in interpreting scaled data for mechanistic conclusions. The major conclusions in these figures that are most relevant are those associated with those origins that are positively regulated by the Fkh1-FHA domain (these are the origin groups most likely to be directly regulated by Fkh1-FHA domain). We have substantially reduced the text associated with these figures, and deemphasized conclusions about additional peaks in the 80 ± 10 bp range outside of the ORC site for origins that were negatively regulated by the Fkh1-FHA domain. While these may be relevant to biology (chromatin opening up in these regions, allowing other factors, not necessarily ORC, to bind and protect the DNA), many more investigations are required to explore this issue. Extensive discussion of the rationale and changes we made as well as additional controls are included in our response to Rev #2.

**Rev #3 Comment #9.** *The data in figure 6 suggest that the FHA domain stabilizes nucleosomes in G1 and destabilizes them in other phases of the cell cycle (based on the consistent observation that nucleosome:sub-nucleosome ratios are lower in G1 cells and somewhat higher in cycling cells, which are a mixed population that includes G1). This effect is observed at TSS and origins – i.e. essentially all well-positioned nucleosomes. The authors appear to disregard the apparently increased nucleosome stability in cycling cells. I am surprised that the strong likelihood of this being an indirect effect is only briefly considered: these data seem like a stray observation to me.*

While we agree that we did not delve into these observations beyond reporting them and briefly discussing them, we disagree strongly that these observations represent a “stray” (aka irrelevant) observation. We also do not agree that there is necessarily a “*strong likelihood of [these phenomenon] being an indirect effect.*” It is possible, for example, that the Fkh1-FHA domain plays a global role in chromosome mobility or positioning within the nucleus (i.e. “higher-order” chromosomal organization, as proposed by the Aparicio lab), and alterations to this higher-order structure in turn alter nucleosome stability. An indirect effect, such as expression defects in a protein that regulates membrane permeability in a cell-cycle independent manner, while less mechanistically applicable, is still important to understand and define.

The direct role (or potential direct role) for Fkh1 at several selected origins has been established through FKH site additions converting non-Fkh1-regulated into Fkh1-regulated origins, and in other published molecular genetic studies, including ours. We did mention the possibility that the effect observed in G1-arrested cells on reduced nucleosome stability may arise from an unknown, downstream effect of Fkh1. We now intentionally discuss the increased nucleosome stability in proliferating cells, which we inadvertently ignored in our first submission. We disagree that these observations are “stray”—whether the effect is direct or indirect, it is a clear molecular phenotype linked to the *fkh1-R80A* genotype.

Given that the MNase data in this study represent the first attempt at this type of high-resolution analyses of Fkh1’s role in chromatin structure, our view was that it was important to share these outcomes, even if (or especially because) they raise the specter of much broader effects of this protein on chromatin that extend well beyond origins and that are poorly understood.

*5. Various aspects of the methods, including the Sort-seq, are inadequately described. It is also not clear to me why the authors apparently did not use pre-existing Sort-seq data analysis pipelines*

We did use the pre-existing pipelines for SortSeq data analyses. The only substantive difference is that we plotted mean values of replicates ± 95% variation. This plotting allowed us to be fully transparent about the variation we observed. We have clarified and better emphasized these points in the Methods section. We hope that the extensive responses above and additional data supplied for the reviewers clarify the study. We thank this reviewer for calling out the distracting nature of the “spikes” in the data. We agree that they added an unnecessary complication to comprehending the study and grasping the major conclusions.

**References**

1.	[Hoggard T, Hollatz AJ, Cherney RE, Seman MR, Fox CA. The Fkh1 Forkhead associated domain promotes ORC binding to a subset of DNA replication origins in budding yeast. Nucleic Acids Res. 2021;49: 10207–10220. doi:](http://paperpile.com/b/0ehuyH/SgNo)[10.1093/nar/gkab450](http://dx.doi.org/10.1093/nar/gkab450)

2.	[Darieva Z, Pic-Taylor A, Boros J, Spanos A, Geymonat M, Reece RJ, et al. Cell cycle-regulated transcription through the FHA domain of Fkh2p and the coactivator Ndd1p. Curr Biol. 2003;13: 1740–1745. doi:](http://paperpile.com/b/0ehuyH/5hA4)[10.1016/j.cub.2003.08.053](http://dx.doi.org/10.1016/j.cub.2003.08.053)

3.	[Reynolds D, Shi BJ, McLean C, Katsis F, Kemp B, Dalton S. Recruitment of Thr 319-phosphorylated Ndd1p to the FHA domain of Fkh2p requires Clb kinase activity: a mechanism for CLB cluster gene activation. Genes Dev. 2003;17: 1789–1802. doi:](http://paperpile.com/b/0ehuyH/mJ69)[10.1101/gad.1074103](http://dx.doi.org/10.1101/gad.1074103)

4.	[Aparicio OM. Location, location, location: it’s all in the timing for replication origins. Genes Dev. 2013;27: 117–128. doi:](http://paperpile.com/b/0ehuyH/H9LQ)[10.1101/gad.209999.112](http://dx.doi.org/10.1101/gad.209999.112)

5.	[Chacin E, Reusswig K-U, Furtmeier J, Bansal P, Karl LA, Pfander B, et al. Establishment and function of chromatin organization at replication origins. Nature. 2023;616: 836–842. doi:](http://paperpile.com/b/0ehuyH/kTeV)[10.1038/s41586-023-05926-8](http://dx.doi.org/10.1038/s41586-023-05926-8)

6.	[Wei W, Hennig BP, Wang J, Zhang Y, Piazza I, Pareja Sanchez Y, et al. Chromatin-sensitive cryptic promoters putatively drive expression of alternative protein isoforms in yeast. Genome Res. 2019;29: 1974–1984. doi:](http://paperpile.com/b/0ehuyH/22Wh)[10.1101/gr.243378.118](http://dx.doi.org/10.1101/gr.243378.118)

7.	[Belsky JA, MacAlpine HK, Lubelsky Y, Hartemink AJ, MacAlpine DM. Genome-wide chromatin footprinting reveals changes in replication origin architecture induced by pre-RC assembly. Genes Dev. 2015;29: 212–224. doi:](http://paperpile.com/b/0ehuyH/EkbL)[10.1101/gad.247924.114](http://dx.doi.org/10.1101/gad.247924.114)

8.	[Speck C, Chen Z, Li H, Stillman B. ATPase-dependent cooperative binding of ORC and Cdc6 to origin DNA. Nat Struct Mol Biol. 2005;12: 965–971. doi:](http://paperpile.com/b/0ehuyH/8imJ)[10.1038/nsmb1002](http://dx.doi.org/10.1038/nsmb1002)

9.	[Feng X, Noguchi Y, Barbon M, Stillman B, Speck C, Li H. The structure of ORC-Cdc6 on an origin DNA reveals the mechanism of ORC activation by the replication initiator Cdc6. Nat Commun. 2021;12: 3883. doi:](http://paperpile.com/b/0ehuyH/wRzM)[10.1038/s41467-021-24199-1](http://dx.doi.org/10.1038/s41467-021-24199-1)

10.	[Zhang H, Petrie MV, He Y, Peace JM, Chiolo IE, Aparicio OM. Dynamic relocalization of replication origins by Fkh1 requires execution of DDK function and Cdc45 loading at origins. Elife. 2019;8. doi:](http://paperpile.com/b/0ehuyH/2An3)[10.7554/eLife.45512](http://dx.doi.org/10.7554/eLife.45512)

11.	[Batrakou DG, Müller CA, Wilson RHC, Nieduszynski CA. DNA copy-number measurement of genome replication dynamics by high-throughput sequencing: the sort-seq, sync-seq and MFA-seq family. Nat Protoc. 2020;15: 1255–1284. doi:](http://paperpile.com/b/0ehuyH/h179)[10.1038/s41596-019-0287-7](http://dx.doi.org/10.1038/s41596-019-0287-7)

12.	[Hawkins M, Retkute R, Müller CA, Saner N, Tanaka TU, de Moura APS, et al. High-resolution replication profiles define the stochastic nature of genome replication initiation and termination. Cell Rep. 2013;5: 1132–1141. doi:](http://paperpile.com/b/0ehuyH/5hdU)[10.1016/j.celrep.2013.10.014](http://dx.doi.org/10.1016/j.celrep.2013.10.014)

13.	[Kwan EX, Wang XS, Amemiya HM, Brewer BJ, Raghuraman MK. rDNA Copy Number Variants Are Frequent Passenger Mutations in Saccharomyces cerevisiae Deletion Collections and de Novo Transformants. G3 . 2016;6: 2829–2838. doi:](http://paperpile.com/b/0ehuyH/5YsR)[10.1534/g3.116.030296](http://dx.doi.org/10.1534/g3.116.030296)

14.	[Yoshida K, Bacal J, Desmarais D, Padioleau I, Tsaponina O, Chabes A, et al. The histone deacetylases sir2 and rpd3 act on ribosomal DNA to control the replication program in budding yeast. Mol Cell. 2014;54: 691–697. doi:](http://paperpile.com/b/0ehuyH/3tXy)[10.1016/j.molcel.2014.04.032](http://dx.doi.org/10.1016/j.molcel.2014.04.032)

15.	[Kwan EX, Foss EJ, Tsuchiyama S, Alvino GM, Kruglyak L, Kaeberlein M, et al. A natural polymorphism in rDNA replication origins links origin activation with calorie restriction and lifespan. PLoS Genet. 2013;9: e1003329. doi:](http://paperpile.com/b/0ehuyH/KwoC)[10.1371/journal.pgen.1003329](http://dx.doi.org/10.1371/journal.pgen.1003329)

16.	[Foss EJ, Lao U, Dalrymple E, Adrianse RL, Loe T, Bedalov A. SIR2 suppresses replication gaps and genome instability by balancing replication between repetitive and unique sequences. Proc Natl Acad Sci U S A. 2017;114: 552–557. doi:](http://paperpile.com/b/0ehuyH/Jr5C)[10.1073/pnas.1614781114](http://dx.doi.org/10.1073/pnas.1614781114)

17.	[Kwan EX, Alvino GM, Lynch KL, Levan PF, Amemiya HM, Wang XS, et al. Ribosomal DNA replication time coordinates completion of genome replication and anaphase in yeast. Cell Rep. 2023;42: 112161. doi:](http://paperpile.com/b/0ehuyH/lA5p)[10.1016/j.celrep.2023.112161](http://dx.doi.org/10.1016/j.celrep.2023.112161)

18.	[Dummer AM, Su Z, Cherney R, Choi K, Denu J, Zhao X, et al. Binding of the Fkh1 Forkhead Associated Domain to a Phosphopeptide within the Mph1 DNA Helicase Regulates Mating-Type Switching in Budding Yeast. PLoS Genet. 2016;12: e1006094. doi:](http://paperpile.com/b/0ehuyH/9IeQ)[10.1371/journal.pgen.1006094](http://dx.doi.org/10.1371/journal.pgen.1006094)

19.	[Yabuki N, Terashima H, Kitada K. Mapping of early firing origins on a replication profile of budding yeast. Genes Cells. 2002;7: 781–789. doi:](http://paperpile.com/b/0ehuyH/LWOK)[10.1046/j.1365-2443.2002.00559.x](http://dx.doi.org/10.1046/j.1365-2443.2002.00559.x)
